# Personal values in soldiers after military deployment: associations with mental health and resilience

**DOI:** 10.3402/ejpt.v5.22939

**Published:** 2014-05-05

**Authors:** Peter Zimmermann, Susanne Firnkes, Jens T. Kowalski, Johannes Backus, Stefan Siegel, Gerd Willmund, Andreas Maercker

**Affiliations:** 1 German Armed Forces Center for Military Mental Health, Berlin, Germany; 2 German Armed Forces 1st Army Division, Hannover, Germany; 3 Institute of Clinical Psychology, Psychopathology and Clinical Intervention, University of Zürich, Zürich, Switzerland

**Keywords:** Beliefs/values, war, logistic regression, treatment readiness, active duty soldiers

## Abstract

**Background:**

After military deployment, soldiers are at an increased risk of developing posttraumatic psychiatric disorders. The correlation of personal values with symptoms, however, has not yet been examined within a military context.

**Method:**

Schwartz’s Portrait Values Questionnaire (PVQ), the Posttraumatic Stress Diagnostic Scale (PDS), and the 11-item version of the Resilience Scale (RS-11) were completed by 117 soldiers of the German Armed Forces who had recently been deployed to Afghanistan (*n=*40 undergoing initial psychiatric treatment, *n=*77 untreated).

**Results:**

Logistic regression showed that the value types of hedonism (−), power (−), tradition (+), and universalism (+) were significantly correlated with the probability and severity of PTSD and whether the participant was in treatment or not. The effects were partially mediated by the RS-11 scale values.

**Conclusions:**

Value types seem to be associated with psychiatric symptoms in soldiers after deployment. These results could contribute to the further development of therapeutic approaches.

In recent years, German Armed Forces (Bundeswehr) soldiers have increasingly sought treatment in Bundeswehr hospitals for deployment-related psychiatric disorders. The most common diagnosis is posttraumatic stress disorder (PTSD) (Kowalski et al., 2012; Zimmermann, Hahne, & Ströhle, 2009). There is also an increased risk for other symptoms in the military, such as alcohol dependence or anxiety (Wittchen et al., 2012). The probability of developing a certain disorder and the severity of symptoms depend on the distribution of individual risk and resilience factors as well as on the distribution of stressful experiences during deployment, which have shown to have discriminant validity when it comes to symptoms (Dias, Sales, Cardoso & Kleber, 2014; Pietrzak, Whealin, Stotzer, Goldstein, & Southwick, 2011).

Risk and resilience factors can include characteristics of the values and norms of traumatized persons (Litz et al., 2009; Siegel & Zimmermann, 2010), although this dimension has not yet been included as complementary diagnostic criteria for PTSD in either the ICD-10 or the DSM-V.

Research on this topic has been very limited. Thus far, studies have focused on associations between posttraumatic symptoms and personal values in victims of crime and people with adjustment disorders following critical life events. The results have shown intercultural variations (Maercker et al., 2009; Müller, Forstmeier, Wagner, & Maercker, 2011). Additional data are available on the impact of values on addictions (Dollinger & Kobayashi, 2003; Galdós & Sánchez, 2010).

No empirical studies on personal value orientations and psychiatric symptoms have been conducted within the military, even though the well-defined and time-limited stressors of out-of-area deployment provide ideal scientific conditions.

The small number of available studies is surprising, considering that the concepts of individual values and their effects on attitudes and behavioral patterns have been a matter of scientific interest since the 1950s (Kluckhohn, 1951; Rokeach, 1973). Existing approaches have gained new impulses from Schwartz’s work and from his “Theory of Basic Human Values” (Schwartz, 1992). Schwartz described values as: “desirable, trans-situational goals, varying in importance, that serve as guiding principles in the life of a person or other social entity.” On the basis of associated motivational goals, he defined 10 interrelated value types (Table 1). These values feature either corresponding or contradictory motivations that are connected with specific consequences for activities. Interest in change and new experiences, which is an aspect of the “stimulation (ST)” value, will most likely *not* go hand in hand with “tradition (TR)” and its associated interest in conservation of customs and habits. “Conformity” and “TR,” in contrast, both lead to an increased desire to fulfill external expectations. Such considerations have led to a circumplex model of values, representing a motivational continuum (Fig. 1) in which adjacent values are similar, while opposite values tend to contrast each other (Hinz, Brähler, Schmidt, & Albani, 2005).

**Fig. 1:**
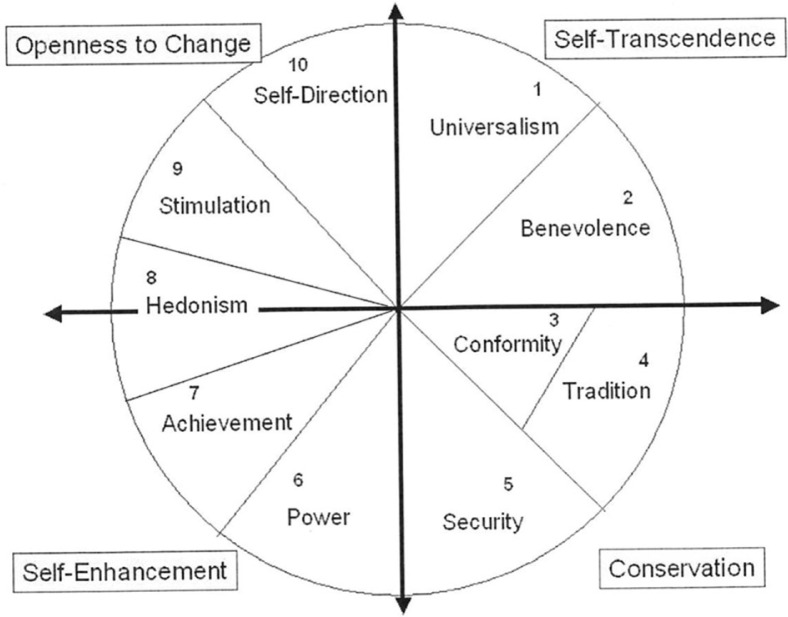
Circumplex structure of personal values based on Schwartz et al., 2001.

**Table 1:** Definition and scale means (M) of value types

Universalism *M*: 2.96 (0.88)	Understanding, appreciation, tolerance, and protection for the welfare of other people
Benevolence *M*: 3.66 (0.69)	Preservation and enhancement of the welfare of people with whom one is in frequent personal contact
Conformity *M*: 3.28 (0.82)	Restraint of actions, inclinations, and impulses likely to upset or harm others and violate social expectations or norms
Tradition *M*: 2.38 (0.72)	Respect, commitment, and acceptance of customs and ideas that traditional culture or religion provide the self
Security *M*: 3.35 (0.85)	Safety, harmony and stability of society, of relationships and of self
Power *M*: 2.57 (1.05)	Social status and prestige, control or dominance over people, and resources
Achievement *M*: 3.16 (1.06)	Personal success through demonstrating competence according to social standards
Hedonism *M*: 3.27 (1.09)	Pleasure and sensuous gratification for oneself
Stimulation *M*: 2.77 (1.07)	Excitement, novelty, and challenge in life
Self-Direction *M*: 3.63 (0.73)	Independent thought and action; choosing, creating, exploring

*Note*. Adapted from the basic human values theory by Schwartz et al. (2001). *M*, mean values of the value types in the sample and (Standard Deviation).

More than 200 studies in 60 countries have confirmed the 10 value types and their circumplex structure (Schwartz et al., 2001). There have also been several approaches in which individual values have been grouped. Schwartz had already divided the values into four second-order value types (Fig. 1): openness to change; self-enhancement; self-transcendence; and conservation (Schwartz, 2003). Maercker suggested differentiating between “traditional” and “modern” values and found discriminant validity for both groups in the severity of symptoms of PTSD (Maercker et al., 2009; Müller et al., 2011).

Genetic and environmental factors have been identified in the development of different values. The question of whether values represent a time-stable disposition to personality structures or whether they are subject to environmental influences, such as military deployments, has not been finally answered, however (Schermer, Feather, Zhu, & Martin, 2008). The test–retest stability of value assessment has generally been high in recent studies but has also shown a sensitivity to life events that has had a limited yet significant impact on value changes. This effect has been greater than the impact of age (Bardi, Lee, Hofmann-Towfigh & Soutar, 2009).

The availability and acceptance of social support as well as the capability or willingness to articulate emotions or talk about symptoms could be possible links between values and the development of psychiatric symptoms, such as adjustment disorders, grief reactions, and PTSD (Brewin, Andrews, & Valentine, 2000; Maercker et al., 2009; Müller et al., 2011).

Furthermore, personal resilience, which has been examined in recent studies as a moderating variable for mental health in the military and in civilian life, might also be associated with values in this regard (Maercker, Gäbler, Schützwohl, O’Neil, & Müller, 2013; Sleijpen, June ter Heide, Mooren, Boeije, & Kleber, 2013; Streb, Häller, & Michael, 2013). According to Werner (1993, 2012) as well Noeker and Petermann (2008), resilient people are able to adapt to adverse, often traumatic environments, resulting in healthy long-term psychological functioning and better developmental outcomes. Resilience is enhanced by adaptive systems on four different levels: personal competencies (coping strategies, self-regulation, motivation, learning); family systems (attachment, parent–child interaction, parenting); resources of social networks (school, peers); and society and culture (norms, values) (Hampel & Petermann, 2006).

Outside the military, personal values have started to be integrated into psychotherapeutic methods over the last few years, and study results have also provided insight into the role of values in the development of diseases. Fegg et al. (2010) were able to strengthen personal values in clinical patients with carcinoma, who subsequently reported an increased sense of meaning in life and quality of life. Additional findings have suggested that a value-based psychotherapeutic approach could facilitate the development of emotional self-regulation (Fujita & Han, 2009).

Within the Bundeswehr, efforts have recently been made to incorporate changes in values and feelings of guilt and shame into traumatherapeutic methods. Pilot studies have indicated that, for patients with deployment experience, behavioral group therapy seems to be an appropriate setting in which to address these complex issues because group coherence makes it easier to verbalize and endure associated emotions (Alliger-Horn & Zimmermann, 2010).

A prerequisite for any treatment, however, is the interest of soldiers in receiving help, which seems to be dependent on attitudes toward mental health services and unit stigma (Brown, Creel, Engel, Herell, & Hoge, 2011). These factors might also be dependent on personal values. The issue is especially relevant since a recent study has shown that only 50% of interviewed German soldiers were seeking any sort of counseling for PTSD after deployment to Afghanistan and only 20% were in psychiatric treatment (Wittchen et al., 2012).

Therefore, the aim of this study was to determine characteristic value types in Bundeswehr soldiers after deployment to Afghanistan and their correlation with the prevalence and severity of PTSD and on the willingness of soldiers to undergo treatment. Based on the small amount of existing data, it was hypothesized that certain value types would be associated with decreased (modern values) or increased (traditional values) frequency and symptom severity of PTSD in soldiers after deployment. Values are part of the resilience construct and thus resilience might be a mediating variable to explain the associations. A mediation analysis should be performed separately for prevalence and severity of PTSD as both dimensions might lead to different views on the planning of therapeutic resources and processes. As values are also connected to perceived social support and fear of stigma, they might be predictors of interest in psychiatric treatment.

## Method

### Participants

The participants in this study were 117 Bundeswehr soldiers who had returned from their deployment to Afghanistan within the previous 12 months. Power analyses had revealed that 120 participants would be necessary in a multiple linear regression analysis [severity of PTSD] to reach an effect size of *f*=0.4 (large effect, Cohen’s *f2*=0.167) with a probability of 97%. This calculation was based on the hypothesis that three values would be identified as a predictor to increase the explanatory power of the model by 10% in addition to the RS-11 (30%). Nine women and 108 men with an average age of 28.8 years (SD=6.8) participated. Of those 117 soldiers, 77 had not undergone psychiatric treatment since their return. Forty participants were assessed at the very beginning of outpatient or inpatient treatment for deployment-related mental illness at the Bundeswehr hospital in Berlin. PTSD was the primary diagnosis in 24 subjects, while a further six were diagnosed with adjustment disorder, five with depression, two with addictive disorders and three with other psychiatric illness.

### Instruments and procedures

The participants completed the Portrait Values Questionnaire (PVQ) (according to Schwartz, 2003; Schwartz et al., 2001), the Posttraumatic Stress Diagnostic Scale (PDS) (Foa, Cashman, Jaycox, & Perry, 1997), and the 11-item German version of the Resilience Scale (RS-11) (Wagnild & Young, 1993). The questions asked in the testing sessions covered the 4 weeks preceding the evaluation.

The primary diagnoses of the clinical patients were based on clinical interviews with medical doctors or clinical psychologists with at least 5 years of training in clinical psychiatry in accordance with the WHO International Classification of Diseases, tenth edition (ICD-10). The diagnoses were re-evaluated and confirmed by two study researchers based on the patient charts. As some of the first study participants expressed concerns that they might be recognizable by their answers, detailed socio-biographical information, such as rank, unit and education, was no longer collected in order to prevent aggravation or dissimulation of symptoms. Types of trauma exposure were also *not* determined in this study.

In 40 items, the PVQ (Schwartz et al., 2001, 2003) measures the degree to which each one of 10 basic values (Table 1) is present, with two to six items attributed to each value type. In two sentences each, these items briefly portray a fictional person and outline a perception or action that is associated with a particular value. Respondents can rate their similarity to the person described on a scale from 1 (not similar at all) to 6 (very similar). The responses to the portraits of each value type are averaged. The PVQ attained a Cronbach’s alpha of 0.55 (median of the value scales) in the validation studies (Schwartz, 2003; Schwartz et al., 2001), in other investigations it reached 0.70–0.74 (Maercker et al., 2009). In this study sample, the median Cronbach’s alpha was 0.54 (median of the value scales). It was used despite this low level because most of the subscales showed acceptable or good values. Test–retest reliability fell in the range of 0.66–0.88. Pearson’s correlation between Schwartz Value Survey (SVS) and PVQ was 0.95 (*p<*0.001).

The PDS was developed by Foa in the early 1990s as a screening instrument for diagnosing PTSD with high specificity (82% consistency with the Structured Clinical Interview of Diagnoses (SCID) diagnostic interview for the Diagnostic and Statistical Manual of Diseases (DSM)). The cut-off for detecting PTSD used in this study was 27 on the global score. The severity of symptoms is measured both in an overall score as well as in subscores relating to the symptom clusters of intrusion, hyperarousal, and avoidance. In evaluation studies, it yielded satisfactory results in terms of test quality with a Cronbach’s alpha of 0.92 and a test–retest reliability of *kappa*=0.74 (Foa et al., 1997).

The 11-item version of the RS by Wagnild and Young (1993) was translated into German in 2004 (RS-11). The original version with 25 items included two subscales that covered the fields of “Personal Competence” (self-reliance, independence, mastery, resourcefulness, perseverance) and “Acceptance of Self and Life” (adaptability, tolerance, a flexible perception of the self, and life). In the German short version, these items were condensed into 11 items in a unidimensional model without loss of validity (Schumacher, Leppert, Gunzelmann, Strauß, & Brähler, 2004). Evaluation by means of a population-based sample (*N*=2,031) showed item-total correlations ranging from *r*_*is*_=0.50 to 0.75 and good internal consistency, with a Cronbach’s alpha of 0.91 (Schumacher et al., 2004).

### Statistical analysis

Schwartz’s 10 value types were the primary explanatory variables of the study. The normal distribution assumption had to be confirmed before parametric evaluation methods could be applied. The Kolmogorov-Smirnov test did not show any abnormalities in the residuals of the 10 value types (i.e., after deduction of the group average values); it only revealed significant scores for self-direction (SD) and universalism (UN) (*p=*0.022 and 0.032, respectively), which implied a possible non-normal distribution. Deviation from normal distribution, however, was not high, so normal distribution was assumed for all of the value types in the following analysis.

Multiple logistic regression was performed in the whole sample (including both treatment and non-treatment groups) with the response variable of presence of PTSD in the PDS scale, while multiple linear regression was performed with the response variable of PTSD severity. Value types, RS-11 total score, age, and sex were explanatory variables. In a step-by-step approach, value types (in the order of their predictive value) and RS-11 total score were successively incorporated into the model. Afterwards a mediation analysis was performed with the RS-11 sum scale values as mediator and value types as predictor. Standardization followed MacKinnon and Dwyer (1993). For indirect effects the Sobel test was used.

The average age of the group that underwent treatment differed significantly from that of the group that did not undergo treatment (*t*(115)=3.21; *p=*0.002). In addition, age had a significant impact on the soldiers’ attitudes towards ST, UN, benevolence (BE) and security (SE). As a consequence, age had to be controlled for in the main evaluations. The results were also controlled for sex.

Multivariate analysis of variance was performed to compare the treatment group and the non-treatment group.

All of the participants provided informed consent. The study was approved by the ethics committee of the Charité University Berlin (no. EA1/203/13).

## Results

The predominant value types of the whole sample were BE (*M*=3.66), SD (*M*=3.63), SE (*M*=3.35), conformity (*M*=3.28), and hedonism (HE) (*M*=3.27) (Table 1).

Table 2 shows the Pearson correlations between the value types and age, resilience and PTSD severity.

**Table 2:** Correlations between value types and age, resilience and PTSD severity

Value type	Age	RS-11	PTSD severity
UN	0.24**	0.13	0.08
BE	0.26**	0.03	.09
CO	0.17	0.08	0.10
TR	0.17	−.04	0.16
SE	0.27**	0.12	0.07
PO	−0.03	0.34**	−0.26**
AC	−0.07	0.32**	−0.19*
HE	−0.18	0.43**	−0.43**
ST	−0.33**	0.30**	−0.25**
SD	0.11	0.40**	−0.00

*Note*. Pearson coefficient

* *p*<0.05

** *p*<0.01. UN, universalism; BE, benevolence; CO, conformity; TR, tradition; SE, security; PO, power; AC, achievement; HE, hedonism; ST, stimulation; SD, self-direction.

The regression model used to predict the *probability of PTSD*, developed from the value types while taking into account age and sex (Table 3), showed power (PO), HE (both negative (−) correlation), and UN (positive (+) correlation) to have significant effects. This model classified 83.5% of the subjects correctly as to the presence of PTSD. The variables UN and TR showed a strong correlation (Pearson’s *r=*0.60, *p<*0.001). In fact, TR could have replaced UN in the model without a significant decrease in the quality of the model. If both UN and TR were included in the model at the same time, however, neither was significant.

**Table 3:** Regression model for probability of PTSD

Variable	*R*	OR	*p*
Results without RS-11 (Cox-Snell-*r* ^2^=0.235)			
Universalism (UN)	0.84	2.32	0.024
Power (PO)	−0.66	0.52	0.029
Hedonism (HE)	−0.72	0.48	0.010
Results incl. RS-11 (Cox-Snell-*r* ^2^=0.338)			
RS-11	−0.12	0.89	<0.001
Universalism (UN)	1.09	2.97	0.014
Power (PO)	−0.62	0.54	0.046

*Note*. Values controlled for age and sex (both always n.s.; not shown). OR, odds ratio; *R*, regression coefficient.

If the RS-11 was included in the model, HE was no longer significant but UN (+) and power (−) remained significant. A total of 86.2% of the subjects were correctly classified, thus making the model that takes resilience into account better than the one that did not include RS-11.

A mediation analysis (Baron & Kenny, 1986; MacKinnon & Dwyer, 1993) with UN, PO, and HE as predictors and resilience as mediator (controlled for age and sex) showed that the effect of UN on PTSD probability was mostly direct, the correlation of PO was roughly two thirds direct, while the one of HE was equal parts directly and indirectly (Table 4).

**Table 4:** Results of mediation analysis for probability of PTSD

Variable	Direct effect	Predictor on mediator	Indirect effect	Total effect
Universalism (UN)	1.13	0.37	−0.04	0.84
(standardized)	0.42		−0.01	0.36
	(*p=*0.012)	(*p=*0.760)	(*p=*0.760)	(*p=*0.024)
Power (PO)	−0.49	2.20	−0.25	−0.66
(standardized)	−0.18		−0.09	−0.29
	(*p=*0.135)	(*p=*0.037)	(*p=*0.067)	(*p=*0.029)
Hedonism (HE)	−0.40	3.78	−0.42	−0.72
(standardized)	−0.150		−0.16	−0.31
	(*p=*0.191)	(*p=*0.001)	(*p=*0.009)	(*p=*0.010)

*Note*. Mediator: RS-11; effect of mediator on outcome: −0.112; standardized: −0.042; *p*<0.001; values controlled for age and sex (both always n.s.; not shown); standardization after MacKinnon & Dwyer, 1993; Sobel test used for indirect effects.

The *severity of PTSD* (Table 5) was significantly correlated with HE (−). With RS-11 included in the model, the significance of HE was retained, with an additional significant effect of SD (+). Again, the explanatory power of the model was increased by the inclusion of resilience.

**Table 5:** Regression model for severity of PTSD

Variable	*R*	*T*	*p*
Results without RS-11 (*r* ^2^=0.20)			
Hedonism (HE)	−0.32	−4.56	<0.001
Results incl. RS-11 (*r* ^2^=0.43)			
RS-11	−0.04	−6.17	<0.001
Hedonism (HE)	−0.21	−3.13	0.002
Self-direction (SD)	0.33	3.39	0.001

*Note*. Values controlled for age and sex (both always n.s.; not shown). *R*, regression coefficient.

Mediation analysis showed that the effect of HE on PTSD severity was almost equal parts direct and indirect, while the effect of SD was *inconsistent*: direct and indirect effects operated in opposite directions (Table 6).

**Table 6:** Results of mediation analysis for severity of PTSD

Variable	Direct effect	Predictor on mediator	Indirect effect	Total effect
Hedonism (HE)	−0.21	3.62	−0.15	−0.36
	(*p=*0.002)	(*p<*0.001)	(*p=*0.001)	(*p<*0.001)
Self-direction	0.33	4.37	−0.187	0.16
(SD)	(*p=*0.001)	(*p=*0.002)	(*p=*0.005)	(*p=*0.158)

*Note*. Mediator: RS-11; effect of mediator on outcome: −0.041; *p*<0.001; values controlled for age and sex (both always n.s.; not shown); Sobel test used for indirect effects.

The differences in value orientations established between the group receiving initial treatment and the untreated group were, on the whole, significant (Pillai’s trace: *F*(10.104)=2.72; *p=*0.005; controlled for age/sex). The two groups also differed significantly in resilience scores on the RS-11 scale, with the group not in therapy achieving a higher score (*M*=63.52; SD=7.47) than the therapy group at the beginning of treatment (*M*=50.51; SD=12.87; *t*(114)=6.88; *p<*0.001). Differences between the groups (after correction of the significance level for 10 post-hoc tests to *p<*0.005) were particularly significant for the value types of power (PO) and HE. The group that did not receive treatment achieved higher scores for these values than the group that did (Table 7).

## Discussion

The objective of this study was to describe the value types of Bundeswehr soldiers after deployment to Afghanistan and to identify associations with the probability and severity of posttraumatic stress as well as with psychiatric treatment readiness. Given the cross-sectional design of the study, the results must be interpreted with caution, but there are currently no empirical studies in which value types and their relationships with mental health were evaluated in a military context.

**Table 7:** Value types of soldiers in psychiatric–psychotherapeutic treatment (1) and of soldiers not in any such treatment (2)

Variable	Group	*M*	SD	Adjusted for age and sex
Universalism (UN)	1	2.86	0.917	*t*(113)=−0.94
	2	3.15	0.789	*p=*0.333
Benevolence (BE)	1	3.59	0.711	*t*(113)=−0.78
	2	3.81	0.644	*p=*0.439
Conformity (CO)	1	3.19	0.842	*t*(113)=−1.10
	2	3.45	0.751	*p=*0.273
Tradition (TR)	1	2.29	0.725	*t*(113)=−1.27
	2	2.55	0.701	*p=*0.206
Security (SE)	1	3.27	0.829	*t*(113)=−0.51
	2	3.50	0.877	*p=*0.609
Power (PO)	1	2.80	0.895	*t*(113)=3.36
	2	2.13	1.178	*p=*0.001
Achievement (AC)	1	3.33	0.949	*t*(113)=2.19
	2	2.85	1.199	*p=*0.031
Hedonism (HE)	1	3.54	0.912	*t*(113)=3.48
	2	2.75	1.210	*p=*0.001
Stimulation (ST)	1	2.98	1.025	*t*(113)=2.07
	2	2.37	1.064	*p=*0.041
Self-direction (SD)	1	3.62	0.700	*t*(113)=0.24
	2	3.64	0.792	*p=*0.812

*Note*. *M*, mean; SD, standard deviation.

The predominant value types of this sample were, starting with the highest mean rating, BE, SD, SE, conformity, and HE (Table 1). A telephone survey of 1,078 participants randomly selected from the German adult population showed different priorities: SD, UN, BE, SE, and HE (Schoen, 2009).

Thus, the results suggest differences in value orientations of soldiers compared to the general population. It remains unclear, however, if the differences are due to Bundeswehr recruitment processes, to military socialization or to deployment-related experiences. This requires further study.

HE was *negatively* correlated with the probability and severity of PTSD in the analysis of the complete study sample. Similarly, power (PO) was negatively correlated with the probability of PTSD. As expected, TR and UN, which lie opposite to HE and power in the circular structure of Schwartz’s value model, had a contrary effect on probability.

Overall, values were weaker than resilience in multivariate prediction of symptom probability and severity. In the mediation analysis, the effect of UN on PTSD probability was mostly direct, the one of PO was roughly two thirds direct, and of HE was equal parts direct and indirect (mediated by RS-11 scale values). The effect of HE on PTSD severity was almost equal parts direct and indirect, while the effect of SD was inconsistent: direct and indirect effects operated in opposite directions.

It seems interesting that probability and severity of PTSD were differently correlated with values. UN and power affected probability but not severity, thus indicating that values might have specific effects in the perception of stress-related symptoms.

All in all, the results concerning SD and power have limited value due to the low Cronbach’s alpha (both 0.54) of these subscales. By contrast, HE and UN had acceptable or good values (0.88/0.75).

The influence of resilience on military-related or civilian posttraumatic symptoms has been proven in numerous studies (Maercker et al., 2013; Streb et al., 2013). It has not yet been investigated, however, how the concepts of values and resilience are connected. Both could represent time-stable personality traits (Noeker & Petermann, 2008; Werner, 2012) that are directly related. In contrast, it can be hypothesized that values might change due to numerous stressful or traumatic life events, such as deployment experiences (Bardi et al., 2009; Wittchen et al., 2012). Longitudinal studies, with measurements before and after deployment, might help clarify this question, but such studies have not been performed to date.

The impact of values as time-stable personality traits on mental health might be explained by subsequent tendencies in how people address mental health issues and how people perceive social support. In accordance with our results, Maercker et al. (2009) found a significant negative correlation between so-called “modern” values (achievement, HE, ST) and the severity of PTSD in German victims of violence. Conversely, “traditional” values (conformity, BE, TR) were linked to an exacerbation of symptoms. The authors explained this finding with a perceived improvement in the social acceptance of victimhood that is associated with these modern values, which results in less fear of isolation and ostracism. Other studies have described mentally ill subjects with traditional values as feeling a stronger sense of social ostracism and guilt about their symptoms (Bennet-Herbert & Dunkel-Schetter, 1992). These associations could explain our study results, too, particularly with respect to HE and TR.

The results, however, have been replicable only to a limited extent. In an elderly Swiss population sample, traditional values were associated with *less* stress resulting from adjustment disorders and grieving processes (Müller et al., 2011). The inconsistency of findings was evident in another study of 275 subjects from various countries, in which the value types of ST and HE and, surprisingly, BE were correlated with the perception of better social support (Goodwin, Costa, & Adonu, 2004).

In addition, the described correlation between values and perceived social support cannot explain why HE and power were *less* pronounced in our study group of subjects receiving psychiatric treatment. A similarly unexpected association in a military context was reported by Brown et al. (2011), who found that greater interest in therapy was associated with higher fear of unit stigma.

Due to these contradictory findings, additional rationales should be discussed: it seems possible that values like UN go hand in hand with more pronounced empathy for the fates of others (Schwartz, 1996) and thus may lead to an increase in suffering from posttraumatic memories and symptoms. A common part of traumatogenic experiences in a military operation is witnessing serious harm to the civilian population but also to fellow soldiers in the course of combat action. In a prevalence study of the Bundeswehr (Wittchen et al., 2012), soldiers deployed to Afghanistan in 2009/2010 reported that they saw destroyed houses and villages (76.4%), encountered injured women and children without being able to help (32%), saw dead and injured fellow soldiers (31.3%, with 1.9% experiencing the incident in question in close proximity), dead bodies and body parts (29.6%) and witnessed violence among the local population (21%). A clear differentiation of deployment experiences, which would have served to correlate them to value types, was, however, not part of this study.

The effect of power might be due to a negative item correlation with PTSD symptoms, as feelings of helplessness and powerlessness are part of the traumatic experience according to DSM-IV and ICD-10 diagnostic classifications. These topics demand further research.

There have been only a few findings that support the hypothesis of a change in personal values due to military deployment. In a civilian study, life events had a significant impact on test–retest reliability of value measurement in a longitudinal assessment of an adult study population (Bardi et al., 2009).

This concept would correspond with the results on Posttraumatic Growth (Tedeschi & Calhoun, 2004). Posttraumatic Growth includes five dimensions that underlie on-going positive changes after traumatic experiences and might show significant overlap with values: greater appreciation of life and a changed sense of priorities, warmer, more intimate relationships with others, greater sense of personal strength, recognition of new possibilities or paths for one’s life and spiritual development.

Although the questions regarding the time-stability of values and the possible impact of external influences, such as military deployment or therapeutic approaches, have not yet been answered, the first clinical experiences have been gathered based on our study results. These experiences have suggested that interventions aimed at UN or TR as personal values and subsequent feelings of failure and related guilt after traumatic stress might have relieving effects and could help patients accept values in line with a critical but fair self-assessment. Thus value orientations might become an important part of psychotraumatological treatment settings in the future (Zimmermann, Biesold, Barre, & Lanczik, 2007). Accordingly, the affirmation of personal values in participants of an experimental study design, using the Trier Social Stress Test, led to a reduced stress reaction, measured as cortisol response compared to controls (Creswell et al., 2005).

While the lack of impact of sex on the statistical model was expected, the results of age were surprising. In international surveys, sex has also been found to have little correlations with value types (Schwartz et al., 2001). Age, however, has been shown to be significantly correlated with the extent to which any one value type was pronounced (Schwartz et al., 2001). The homogenous age structure of this sample with a low rate of female participants might explain the differences.

### Limitations

This study was limited in its predictive power due to its cross-sectional design, which did not allow us to establish clear causal attributions of deployment-related stressors, changes in values, and psychiatric illness. The small sample size was another limitation. Inferences about the prevalence of mental illness were also problematic, as only self-report measurements were obtained and no standardized diagnostic interviews were conducted as part of this study, although the PDS corresponds quite well to such interviews.

The results of this study are not readily applicable to other subject groups, such as policemen and firefighters, because the development of values is closely linked to the subjects’ cultural environment, socio-demographic factors, work environment, and experiences to be processed. Nevertheless, these occupational groups also face extreme work-related stress and their hierarchical structures with predominantly male staff suggest further similarities. To our knowledge, there have not been any studies in these populations either.

## Conclusions

Despite its methodological limitations, this study suggests that there are associations between value types (especially HE, power, TR, and UN) and mental health as well as treatment readiness in soldiers after military deployment. Further research is required to determine other influencing variables, such as perceived stigma and resilience, and whether these results might be applicable to forces with other types of stress, such as police forces and firefighters. Such research should be longitudinal in design, with measurements before and after deployment, and should take the nature of stressful events experienced into account.

As cultural aspects beyond the direct military environment might influence the development of values, other armed forces should also conduct similar studies to allow for comparisons. Such an international comparison could result in additional insights into the development of values and relevant influencing factors.

A possible suggestion based on the basis of the data gathered here would be that addressing values could be beneficial in clinical psychotraumatology settings in the military work environment.
